# Antioxidative Role of Hatikana (*Leea macrophylla* Roxb.) Partially Improves the Hepatic Damage Induced by CCl_4_ in Wistar Albino Rats

**DOI:** 10.1155/2015/356729

**Published:** 2015-06-14

**Authors:** Samina Akhter, Md. Atiar Rahman, Jannatul Aklima, Md. Rakibul Hasan, J. M. Kamirul Hasan Chowdhury

**Affiliations:** Department of Biochemistry and Molecular Biology, University of Chittagong, Chittagong 4331, Bangladesh

## Abstract

This research investigated the protective role of *Leea macrophylla* extract on CCl_4_-induced acute liver injury in rats. Different fractions of *Leea macrophylla* (Roxb.) crude extract were subjected to analysis for antioxidative effects. Rats were randomly divided into four groups as normal control, hepatic control, and reference control (silymarin) group and treatment group. Evaluations were made for the effects of the fractions on serum enzymes and biochemical parameters of CCl_4_-induced albino rat. Histopathological screening was also performed to evaluate the changes of liver tissue before and after treatment. Different fractions of *Leea macrophylla* showed very potent 2,2-diphenyl-1-picrylhydrazyl (DPPH) radical scavenging effect, FeCl_3_ reducing effect, superoxide scavenging effect, and iron chelating effect. Carbon tetrachloride induction increased the level of serum aspartate aminotransferase (AST), alanine aminotransferase (ALT), and alkaline phosphatase (ALP) and other biochemical parameters such as lipid profiles, total protein, and CK-MB. In contrast, treatment of *Leea macrophylla* reduced the serum aspartate aminotransferase (AST), alanine aminotransferase (ALT), and alkaline phosphatase (ALP) activities as well as biochemical parameters activities. *L. macrophylla* partially restored the lipid profiles, total protein, and CK-MB. Histopathology showed the treated liver towards restoration. Results evidenced that *L. macrophylla* can be prospective source of hepatic management in liver injury.

## 1. Introduction

The continuous and varied exposure of liver to xenobiotics often makes it absorb toxins from the intestinal tract resulting in a variety of hepatic damage which is associated with distortion of many metabolic functions regulated by liver [1, 2]. Liver damage ranges from acute infectious diseases to hepatoma, through cellular death, inflammation, immune response, fibrosis, ischemia, and altered gene expression. Steroids, vaccines, and other conventional or synthetic drugs which have been employed to treat these liver diseases are inadequate and sometimes can have potential adverse effects especially when administered in long term. Sometimes they are highly expensive and not much effective. Presently, the use of herbal medicines for prevention and control of chronic liver diseases is in the focus of attention for the physicians, pharmaceutical manufacturers, and patients; this shift towards the herbal drugs is due to the effectiveness, less side-effects, and low cost of herbal medicines. Plant-derived drugs are also high potentials for scavenging the reactive oxygen and nitrogen species produced in most of the hepatic complications that involve hepatocyte, Kupffer, stellate, and endothelial cells [3]. Plant-derived drugs, therefore, seem to be very attractive alternative for the healing of hepatic diseases [4, 5].

*Leea macrophylla* (Roxb.), locally known as Hathikana or Hatikana belonging to Leeaceae family, is a herb or herbaceous shrub with a very big size leaf like an elephant-ear [6]. The plant is native to North-Eastern India; however, it is distributed to the relatively hotter parts of India, central and eastern Nepal, Bhutan, China, Myanmar, Thailand, Cambodia, and Laos [7]. Few areas such as Rajshahi, Jessore, and Natore are noteworthy for the habitat of* L. macrophylla* in Bangladesh. Ethnobotanical survey of this plant shows some important therapeutic uses in cancer, dysentery, body-ache, and sexual disability [8]. It has some other traditional uses for tonsillitis, tetanus, nephrolithiasis, rheumatism, arthritis, snake bites, sore, pain, and blood effusion [9, 10]. Recently the plant has been studied for its seed, leaf, and roots. These are extensively used by the ayurvedic physicians in the preparation of seasonal tonic modaka preparation [6]. Leaf juice is recognized as local anti-inflammatory agent and used in boils, arthritis, gout, and rheumatism [11]. Seed extract of this plant revealed the presence of carbohydrate, protein, glycosides, phenolics, and saponin. The extracts also exhibit antifungal activities against* Candida albicans* except the extract n-hexane [12]. Anti-inflammatory and analgesic [13], antinociceptive and cytotoxic [14], and urolithiatic [15] effects of this plant leaf extract have been reported by the scientists. However, isolation and identification of phytochemical constituents of this plant have not been yet evidenced. We have reported the major phytochemical groups present in different fractions of the extract to supplement our major target to investigate the hepatoprotective effect of this plant, how it contributes in protecting the carbon tetrachloride induced hepatic damage in experimental rats.

## 2. Materials and Methods

### 2.1. Chemical and Reagent

Curcumin (≥80%), DMSO (dimethyl-sulfoxide, 99.9%), ferric chloride, hydrogen peroxide (30%, W/W), 1,1-diphenyl-2-picrylhydrazyl (DPPH), NBT (nitro blue tetrazolium), potassium ferric cyanide, sodium hydroxide, and trichloroacetic acid (TCA), obtained from Sigma Aldrich Co., St. Louis, USA, were used for this experiment. All chemicals and reagents were of the analytical grade.

### 2.2. Plant Collection and Authentication

The plant material,* Leea macrophylla* (leaf), was collected from the cultivated area of Bangladesh Council of Scientific and Industrial Research (BCSIR), Rajshahi, during the months of May–August 2013. Plant was identified and authenticated by Dr. Shaikh Bokhtear Uddin, Taxonomist and Associate Professor of the Department of Botany, University of Chittagong, Bangladesh. A voucher specimen (accession number ACCU-2011/07) that contains the identification characteristics of the plant has been preserved for future reference.

### 2.3. Preparation of Plant Extract

Plant samples were washed and air-dried for three weeks to a constant weight and then processed to fine powder using locally made Willy mill before storage in air-tight dry containers until needed. The methanol extract of* Leea macrophylla* was prepared as previously described by Rahman et al. (2013) [16]. Briefly, the powdered plant material (1 kg) was defatted through n-hexane and extracted with 5 L 98% methanol (maceration) for a week at room temperature (23 ± 0.5)°C. The extract was filtered through Whatman filter paper number 1 and concentrated on a rotary evaporator (RE 200, Bibby Sterling Ltd., England) at 40°C under reduced pressure. The concentrated extract was finally evaporated to dryness on a water bath which afforded 196 g of crude extract. The crude methanol extract (196 g) was suspended in 300 mL of distilled water and successively partitioned with chloroform (2 × 300 mL) and ethyl acetate (2 × 300 mL) to obtain a fraction of chloroform extract (19 g) and ethyl acetate extract (21 g) [17]. The concentrated extract was collected in petriplate and allowed to preserve at 4°C until further use.

### 2.4. Optimization of the Extraction Procedure

Established procedure was applied to optimize the crude extraction [18]. Briefly, the dried leaves were put through a process of extraction with methanol 98% to 23 ± 0.5°C. A central composition design was done in order to evaluate the effect of the progress variables: extract time (T: 36 to 72 h) and solvent-seed ratio (SSR: 2/1 to 4/1) expressed as milliliter of solvent per gram of dry leaf. The response variable was as follows: crude extract yielded from each gram of dry powder. The extract was filtered using filter paper (Whatman, Grade 589/2) and the solvent was eliminated using a vacuum evaporator (RE 200, Bibby Sterling Ltd., England).

### 2.5. Qualitative Phytochemical Group Tests

The fractions were subjected to qualitative screening for the detection of phytochemical groups by established methods. In each test 10% (w/v) solution of the extract was taken unless otherwise mentioned in the individual test [19].

### 2.6. DPPH Free Radical Scavenging Assay

DPPH free radical scavenging activity of* Leea macrophylla* extract was determined in comparison to standard antioxidant ascorbic acid. The whole procedure was administrated according to established procedure by Sanja et al., with slight modifications [20]. Specific amount of ascorbic acid was dissolved in methanol to prepare stock solution of 1 *μ*g/mL. Specific amount of ascorbic acid was dissolved in methanol to prepare stock solution of 1 mg/mL. The stock solution was diluted to give the concentration of 30, 60, 120, 240, and 480 *μ*g/mL. Similarly 10 mg of dried different extracts was dissolved in 10 mL of specific solvent for the preparation of stock solution of sample to give concentration of 1 mg/mL. The stock solution was diluted to give the concentrations of 30, 60, 120, 240, and 480 *μ*g/mL. Required amount of DPPH was dissolved in methanol and the solution was kept in conical flax. It was protected from light by covering the flax with aluminum foil.

The scavenging activity against DPPH was calculated using the following equation:

(1) $$\begin{array}{c} \%I=\left\{\;\frac{\left(A_{0}-A_{1}\right)}{A_{0}}\;\right\}\times 100, \end{array}$$

where *A*_0_ is the absorbance of the control (freshly prepared DPPH solution) and *A*_1_ is the absorbance of the extract/standard.

Then the percentage of scavenging activity or inhibition was plotted against log concentration and from the graph IC_50_ value was calculated by linear regression analysis.

### 2.7. Ferric Reducing Effect Assay

The reducing power of* L. macrophylla* leaf extract was determined according to the modified method of Sanja et al. [20]. Sample was prepared at the concentrations of 30, 60, 120, 240, and 480 *μ*g/mL in phosphate buffered saline (pH 6.6). Sample and standard were mixed with 2.5 mL of 1% potassium ferric cyanide. This reaction mixture was incubated at 50°C for 20 min. After incubation reaction mixture was cooled and 2.5 mL of 10% trichloroacetic acid was added followed by proper mixing. It was centrifuged at 3000 rpm for 10 minutes. A 2.5 mL of distilled water was added to 2.5 mL of the supernatant. To this reaction mixture 1 mL of 0.1% ferric chloride was added followed by 10 min incubation at room temperature. Absorbance was measured at 700 nm. Ascorbic acid was used as a standard. The increase of the reducing power by the extract and standard was calculated by the following formula:

(2) Percentage  increase  of  reducing  power=AtestAcontrol−1×100,

where *A*_(test)_ is the absorbance of test solution. *A*_(control)_ is the absorbance of control.

Increased absorbance of the reaction mixture indicates an increase in reducing power.

### 2.8. Superoxide Scavenging Activity Assay

Superoxide scavenging activity of* L. macrophylla *extract was determined by the alkaline DMSO method described by Pandey et al. with slight modification [21]. In this method, the concentration of superoxide in the alkaline DMSO system corresponds to the concentration of oxygen dissolved in DMSO. Briefly, superoxide radical was generated in nonenzymatic system. To the reaction mixture containing 0.1 mL of NBT (1.0 mg/mL solution in DMSO) and 0.3 mL of the extract (50, 100, 200, 400, and 800 *μ*g/mL) and standard (curcumin 5, 10, 20, 40, and 80 *μ*g/mL) in DMSO, 1.0 mL of alkaline DMSO (1.0 mL DMSO containing, 5 mM NaOH in 0.1 mL water) was added to give a final volume of 1.4 mL and the absorbance was measured at 560 nm. Control was prepared by mixing 300 mL of plain DMSO, 0.1 mL NBT solution, and 1.0 mL alkaline DMSO. The decrease in the absorbance at 560 nm with antioxidants indicated the consumption of generated superoxide [22, 23]. The percentage of superoxide radical scavenging by the* L. macrophylla* extract and standard scavenger curcumin was calculated as follows:

(3) Percentage  of  superoxide  scavenging  activity=Absorbancetest−AbsorbancecontrolAbsorbancetest×100.

### 2.9. Iron Chelating Effect Assay

Iron chelating activity was determined according to the method described by Benzie and Strain with slight modification [24]. The principle is based on the formation of O-phenanthroline [Fe^2+^] complex and its disruption in the presence of chelating agents. A 2 mL of test sample and standard ascorbic acid of different concentrations (25, 50, 100, 200, 400, and 800 *μ*g/mL) were taken triplicate of test tubes, respectively. A 1 mL of O-phenanthroline and 2 mL of ferric chloride solution were added. Control was prepared in similar manner excluding sample. Then the reaction mixture was incubated for 10 minutes at room temperature and the absorbance of the reaction mixture was measured at 510 nm by using visible spectrophotometer. The percentage of iron chelating activity of* L. macrophylla* was calculated by the following equation:

(4) Percentage  of  chelating  activity=Test  absorbance−controlTest  absorbance×100.

### 2.10. Experimental Animals and Hepatoprotective Effects

The protocol employed met the guidelines of the Good Laboratory Practice (GLP) regulations of World Health Organization and the rules and regulations of experimental animal ethics committee of Faculty of Biological Sciences, University of Chittagong, were duly followed. Six-to-seven-week-old apparently healthy Wistar Albino rats weighing 100–120 g were obtained from the Department of Pharmacy, Jahangir Nagar University, Savar, Bangladesh. Animals were acclimatized to laboratory condition before the start of experiment. The animals were maintained in polycarbonated cages (23 ± 2°C, relative humidity 60–70%, 12 : 12 h light : dark cycle) and fed on a commercial rat chow with drinking water* ad libitum*. All animal experimentations were maintained and carried out with the institutional animal review board of Faculty of Biological Science, University of Chittagong, Bangladesh.

### 2.11. Experimental Procedure

Forty-five Wistar rats (100–120 g) of both sexes were randomly allocated into nine groups of five rats each in order to investigate the effects of the organic extracts on the carbon tetrachloride induced liver damage. Rats in Group I were uninfected and served as normal control to receive normal food and water* ad libitum *for seven days. Rats in Groups II–IX were each intraperitoneally injected with carbon tetrachloride (1 mL/kg) for seven days. Group II served as hepatic control while all the animals in this group remained untreated. Group III served as reference control and received silymarin (25 mg/kg, orally) for seven days. Groups IV to IX served as treatment groups while rats in these groups received the following oral dosages of* L. macrophylla* for seven days.

Group IV received methanol 100 mg/kg bw, Group V received methanol 200 mg/kg bw, Group VI received chloroform extract 100 mg/kg bw, Group VII received chloroform extract 200 mg/kg bw, Group VIII received ethyl acetate extract 100 mg/kg bw, and Group IX received ethyl acetate extract 200 mg/kg bw. In order to assess organ damage in all the groups of animals, the serum prepared from the blood of all animals after humane decapitation on day 8 after infection (pi) was used to measure the hepatic and relevant markers using commercial reagent kits (Randox Laboratories, Ireland). The liver was excised and fixed in bouin's fluid for histopathological assessment of liver damage.

### 2.12. Acute Toxicity Test

Acclimatized Wistar albino rats maintained in the mentioned laboratory condition were used for acute toxicity study. Five animals received a single oral dose of 0.5, 1.0, 1.5, and 2.0 g/kg BW of each of the fractions of the extract. Animals were kept overnight fasting prior to administration of the extract. After administration, food was withheld for further 3 to 4 h. Individual animal was kept in close observation during the first 30 min after dosing, periodically first 24 h (special attention for the first 4 h), and thereafter for a period of 14 days to record the delayed toxicity. Once daily cage side observation including changes in skin and fur, eyes and mucous membrane, respiratory and circulatory rate, and autonomic and CNS changes were observed. The effective therapeutic dose was taken as one-tenth of the median lethal dose (LD_50_ > 2.0 g/kg) [25].

### 2.13. Assessment of Liver Damage

Separated serum was assessed for the quantitative measurement of total protein (TP), triglyceride (TG), bilirubin, cholesterol (CHL), alkaline phosphatase (ALP), alanine aminotransferase (ALT), aspartate aminotransferase (AST), low density lipoprotein (LDL), high-density lipoprotein (HDL), and creatinine kinase-MB (CK-MB). Cellular morphology of liver was screened by histopathological assessment of liver damage through Haematoxylin and Eosin (H&E) stained slides of liver tissue, including cell necrosis, fatty change, infiltration of kupffer cells, and lymphocytes.

### 2.14. Data Analysis

All data are presented as the Mean ± SD of five animals. Data was analyzed with a statistical software package (SPSS for Windows, Version 21, IBM Corporation, NY, USA) using Tukey's HSD multiple range post hoc test. Values were considered significantly different at *p* < 0.05.

## 3. Results 

### 3.1. Phytochemical Contents of Hatikana

The major phytochemical metabolites present in the extract of* L. macrophylla* are summarized in Table 1.

### 3.2. DPPH Free Radical Scavenging Effect

**Figure 1:**
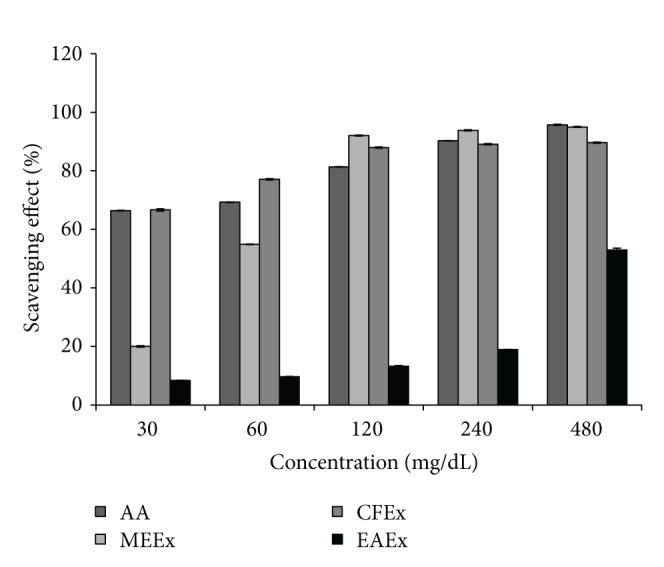
DPPH free radical scavenging effect of different fractions of* L. macrophylla* extract. Data are shown as Mean ± SD for triplicate. Data were analyzed by one-way ANOVA followed by Tukey's post hoc test (SPSS, Version 21.0, NY) for multiple comparisons. Values with *p* < 0.05 were considered significant.

The results for DPPH free radical scavenging effect of the extract shown in Figure 1 indicated that there was a significant (*p* < 0.05) difference of mean percentage of scavenging effect between all the tested concentrations of the extract and reference antioxidant ascorbic acid. Three extracts methanol, chloroform, and ethyl acetate extracts showed the highest scavenging activity 94.97 ± 0.19%, 89.63 ± 0.21%, and 52.98 ± 0.59% at concentration 480 *μ*g/mL which are significant compared to that (95.72 ± 0.17%) of ascorbic acid. The inhibition concentration (IC_50_) of the extract was determined by plotting a graph (Figure 5(a)) of scavenging activity against the log concentration. The IC_50_ values of the methanol, chloroform, and ethyl acetate extracts were found to be 55.46 *μ*g/mL, 2.64 *μ*g/mL, and 946.24 *μ*g/mL while IC_50_ value of chloroform extract was statistically significant (*p* < 0.05) and even lower than that (8.47 *μ*g/mL) of ascorbic acid (Table 2).

### 3.3. FeCl_3_ Reducing Power

**Figure 2:**
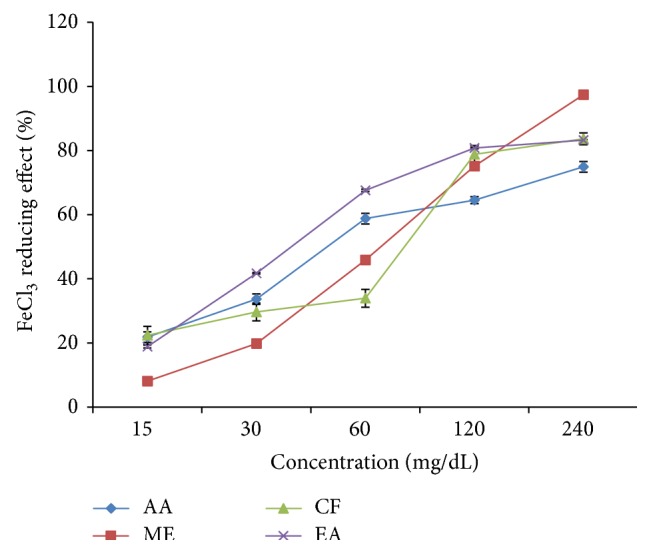
FeCl_3_ reducing power of different fractions of* L. macrophylla* extract. Data are shown as Mean ± SD for triplicate. Data were analyzed by one-way ANOVA followed by Tukey's post hoc test (SPSS, Version 21.0, NY) for multiple comparisons. Values with *p* < 0.05 were considered significant.

*Leea macrophylla* extract and ascorbic acid showed a dose dependent reducing activity (Figure 2) in FeCl_3_ assay. Methanol, chloroform, and ethyl acetate extracts showed the highest reducing power 97.40 ± 0.28%, 83.64 ± 1.81%, and 83.23 ± 0.19%, respectively, at concentration 480 *μ*g/mL. On the other hand ascorbic acid as standard showed the highest reducing power 74.91 ± 1.60% at concentration 16 *μ*g/mL. The percentage (%) of reducing power or % of inhibition was plotted against log concentration and from the graph IC_50_ value was calculated by linear regression analysis (Figure 5(b)). IC_50_ values of ascorbic acid, methanol, chloroform, and ethyl acetate extracts were found to be 7.72 *μ*g/mL, 122.74 *μ*g/mL, 15.24 *μ*g/mL, and 84.91 *μ*g/mL, respectively (Table 2).

### 3.4. Superoxide Radical Scavenging Activity by Alkaline DMSO Method

**Figure 3:**
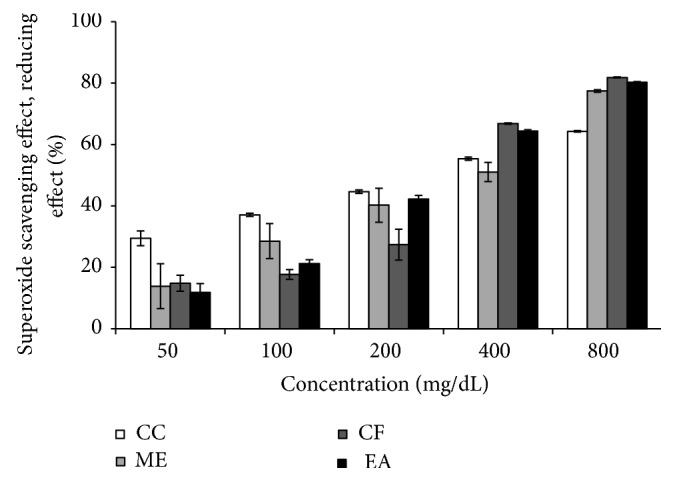
Superoxide radical scavenging effect of different fractions of* L. macrophylla* extract. Data are shown as Mean ± SD for triplicate. Data are shown as Mean ± SD for triplicate. Data were analyzed by one-way ANOVA followed by Tukey's post hoc test (SPSS, Version 21.0, NY) for multiple comparisons. Values with *p* < 0.05 were considered significant.

Superoxide radical was formed by alkaline DMSO which reacted with NBT to produce colored diformazan. The ethanolic extract of* L. macrophylla* scavenges superoxide radical and thus inhibits formazan formation. Superoxide scavenging activity of* L. macrophylla* extract and reference compound curcumin showed a dose dependent activity (Figure 3). Superoxide scavenging activity of curcumin and different extract of* L. macrophylla* showed a dose dependent activity. Among the five different concentrations (5, 10, 20, 40, and 80 *μ*g/mL) curcumin showed the highest scavenging activity was 64.37 ± 0.259% at 80 *μ*g/mL. At the concentration 800 *μ*g/mL, methanol extract of* L. macrophylla* leaf showed the best scavenging activity 77.41 ± 0.44%. Chloroform and ethyl acetate extract of* L. macrophylla* showed 81.82 ± 0.15% and 80.09 ± 0.23% of scavenging activity at the latest concentration. Percentage of scavenging activity or inhibition was plotted against log concentration and IC_50_ value was calculated from linear regression analysis (Figure 5(c)). IC_50_ values of curcumin, methanol, chloroform, and ethyl acetate extract of* L. macrophylla* were found to be 271.02, 287.07, 274.16, and 252.35 *μ*g/mL, respectively.

### 3.5. Iron Chelating Activity

**Figure 4:**
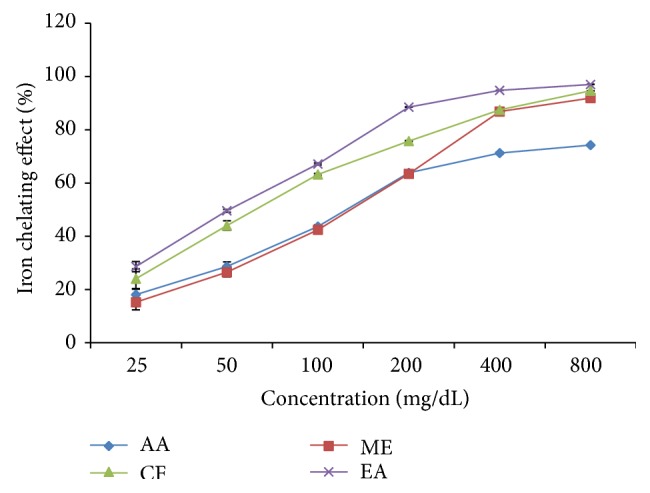
Iron chelating effect of different fractions of* L. macrophylla* extract. Data are shown as Mean ± SD for triplicate. Data were analyzed by one-way ANOVA followed by Tukey's post hoc test (SPSS, Version 21.0, NY) for multiple comparisons. Values with *p* < 0.05 were considered significant.

**Figure 5:**
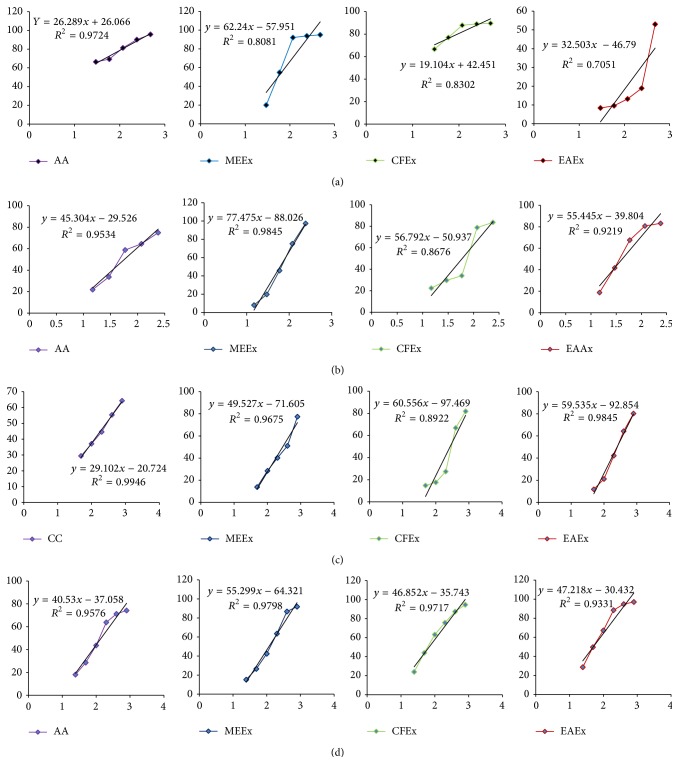
Regression analyses to calculate the IC_50_ values of the fractions of the extract. AA, Ascorbic acid; MEEx, methanol extract; CFEx, chloroform extract; EAEx, ethyl acetate extract; CC, curcumin. (a) DPPH free radical scavenging effect; (b) FeCl_3_ reducing effect; (c) superoxide scavenging effect; (d) iron chelating effect.

The iron chelating effect of* L. macrophylla* extract and standard antioxidant ascorbic acid is shown in Figure 4. The highest chelating activity of ascorbic acid showed 74.20% at concentration 800 *μ*g/mL. On the other hand* L. macrophylla *leaf extract of methanol, chloroform, and ethyl acetate showed the highest chelating activity 91.86 ± 0.01%, 94.59 ± 0.015%, and 97.02 ± 0.006%, respectively, at concentration 800 *μ*g/mL. Percentage of chelating activity was plotted against log concentration and from the graph inhibition concentration value was calculated by linear regression analysis (Figure 5(d)). IC_50_ value of ascorbic acid and* L. macrophylla* leaf extract of methanol, chloroform, and ethyl acetate was found to be 142.23 *μ*g/mL and 118.02 *μ*g/mL, 68.55 *μ*/mL and 51.29 *μ*g/mL, respectively (Table 2).

### 3.6. Acute Toxicity Assay

The crude extract and fractions were found to be nontoxic in acute toxicity test. No mortality or abnormality was recorded in the experiment.

### 3.7. Effect of* L. macrophylla* Extract on ALT, AST, and ALP

**Figure 6:**
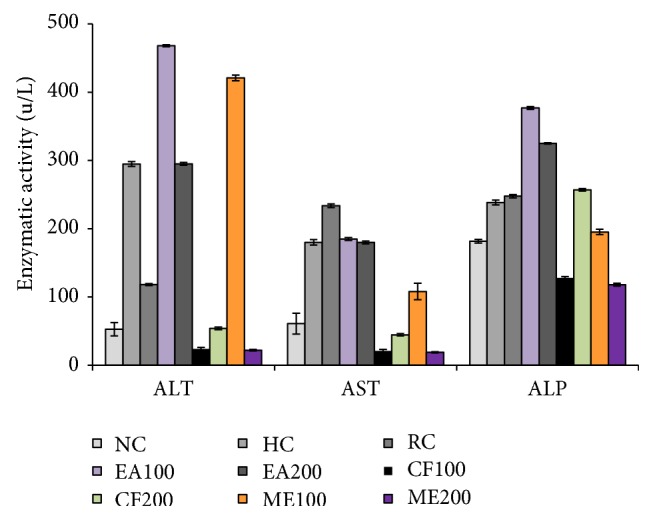
Effect of* L. macrophylla* on enzymatic parameters in CCl_4_ intoxicated Wister rats. Data are shown as Mean ± SD for triplicate. Data are shown as Mean ± SD for triplicate. Data were analyzed by one-way ANOVA followed by Tukey's post hoc test (SPSS, Version 21.0, NY) for multiple comparisons. Values with *p* < 0.05 were considered significant.

Liver marker enzyme ALT was found to be 52.67 ± 9.71 U/L in the animals of normal control group. The administration of CCl_4_ to the hepatic control group resulted in a mark increased in a serum ALT level (294.67 ± 3.5 *μ*/L) which was restored to 24.00 ± 2.00, 54.00 ± 2.00, and 22.00 ± 1.00 *μ*/L with the treatment Groups IV (methanol extract 100 mg/kg bw), VI (chloroform extract 100 mg/kg bw), and VII (chloroform extract 200 mg/kg bw). The values were statistically significant (*p* < 0.05) as compared to Group II (hepatic control group) (Figure 6).

AST level in Group I (normal control group) (6.00 ± 15.39 *μ*/L to) was highly increased to (180.00 ± 4.00 *μ*/L) in hepatic control. This elevated level of serum AST was decreased towards the normalization with the administration of* L. macrophylla* extract. However, treatment Groups V, VI, and VII significantly (*p* < 0.05) reduced the AST levels to 19.00 ± 1.00, 21.00 ± 2.00  *μ*/L, and 44.67 ± 1.53  *μ*/L (Figure 6).

In restoration of ALP level, methanol extract 200 mg/kg showed the highest activity compared to other extracts. Groups IV, V, and VI successfully reduced the serum ALP level towards the normal values of ALP level in serum (Figure 6).

### 3.8. Effect of* Leea macrophylla* Leaf on CHL, HDL, LDL, and TG

**Figure 7:**
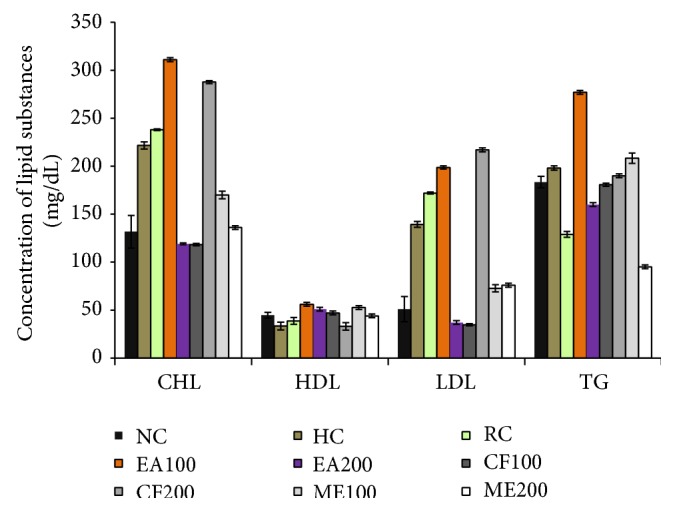
Effect of* L. macrophylla* (leaves) on biochemical parameters of CCl_4_ intoxicated Wister rats. Data are shown as Mean ± SD for triplicate. Data were analyzed by one-way ANOVA followed by Tukey's post hoc test (SPSS, Version 21.0, NY) for multiple comparisons. Values were considered significant with *p* < 0.05.

After assaying hepatic biochemical marker CHL, it was revealed that the level of CHL in normal control group was 131.67 ± 17.01 mg/dL. Injection of CCl_4_ successfully damaged liver and the normal control value increased to 221.67 ± 3.79 mg/dL in hepatic control group. After applying three different extracts of* L. macrophylla*, the level of CHL was greatly reduced to 118.00 ± 1.15 mg/dL by chloroform (100 mg/kg). These levels of CHL in treatment group indicate that* L. macrophylla* successfully decreased serum CHL compared to hepatic control. In restoration of CHL toward normalization chloroform extract (100 mg/dL) showed the most promising effect (Figure 7).

The entire tree extracts of* L. macrophylla* worked for normalizing the HDL level which was reduced in the hepatic damage produced by CCl_4_ injection in normal control group. In particular, Groups V, VI, VIII, and IX restored the HDL level which was significant compared with the normal control (Figure 7).

In the CCl_4_ treated hepatic control group serum LDL level was 139.33 ± 3.06 mg/dL. In normal control group this level was 51.00 ± 13.11 mg/dL. In treatment group, ethyl acetate 200 mg/kg bw and chloroform 100 mg/kg bw decreased the high level of LDL to 37.00 ± 2.00 mg/dL and 34.67 ± 1.15 mg/dL. These values were statistically significant and even lower than the LDL values of normal control group (Figure 7).

With respect to the other biochemical parameters examined here, the present results revealed that administration of CCl_4_ alone did not cause any significant change in biochemical parameters TG. TG level was 183.33 ± 36.02 mg/dL in normal control which was slightly increased in hepatic control group (198.00 ± 2.2 mg/dL). This increased TG level was significantly and promisingly decreased by 200 mg/kg of methanol extract which normalized the TG level 95.00 ± 2.00 mg/dL (Figure 7).

### 3.9. Effect on Total Protein and CK-MB

*Leea macrophylla* also showed an effect on decreasing the total protein and CK-MB in this experiment. Sudden increase of total protein and CK-MB due to the hepatic damage was restored with* L. macrophylla* treatment. Among the treatment groups, chloroform 100 mg/kg bw and methanol extract 200 mg/kg bw significantly restored the increase amount of serum total protein. Serum total protein in normal group (4.63 ± 0.51) was reduced to 3.97 ± 0.15 by chloroform (100 mg/kg bw) and 2.50 ± 0.20 by methanol extract (200 mg/kg bw). Most of the treatment groups except methanol extract (200 mg/kg bw) served as repairing agent helped in normalizing the serum CK-MB level in hepatic damage while ethyl acetate 200 mg/kg bw and chloroform 100 mg/kg bw played the most significant role in restoring the serum CK-MB (Table 3).

### 3.10. Histopathological Screening of the Liver Tissue

**Figure 8:**
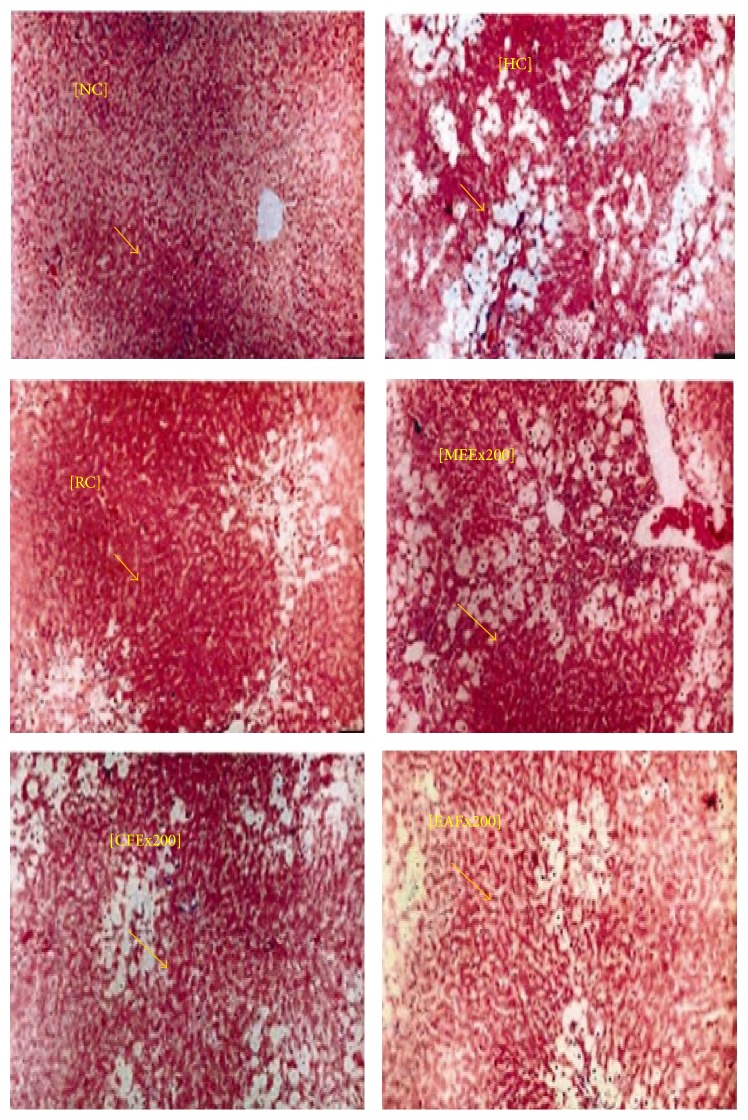
Histopathological examination of liver tissue at the end of the experimental period. NC, normal control; HC, hepatic control; RC, reference control (silymarin treated); MEEx 200, methanol extract 200 mg; CFEx 200, chloroform extract 200 mg; EAEx200, ethyl acetate extract 200 mg. Arrow shows the position of liver tissue containing pancreatic islets.

Histopathological examination of liver tissue showed that the damaged or impaired liver has been partially repaired through the treatment with* L. macrophylla* extracts. Chloroform fraction has given the highest protection to the liver against CCl_4_ (Figure 8).

## 4. Discussions

Polyphenolic compounds present in plant extract significantly contribute to the plant defense mechanism. They also play key roles to the pathophysiological issues of animal as well as human. To evaluate the scavenging effect of the extract in this study, DPPH reduction was investigated against positive control ascorbic acid. The DPPH-stable free radical method is a sensitive way to determine the antioxidant activity of plant extracts [26, 27]. The odd electron in the DPPH free radical gives a strong absorption maximum at 517 nm and is purple in color [28]. The color turns from purple to yellow when the odd electron of DPPH radical becomes paired with hydrogen from a free radical scavenging antioxidant to form the reduced DPPH-H. The resulting decolorization is stoichiometric with respect to the number of electrons captured. The more antioxidants occurred in the extract, the more DPPH reduction occurs.

The quantification of antioxidant in the extract is made by calculating the IC_50_ value. Chloroform fraction among the three fractions showed the lowest IC_50_ value (2.64 *μ*g/mL) which was statistically significant to that of ascorbic acid 8.47 suggesting a high radical scavenging activity of* L. macrophylla.* IC_50_ value of methanol extract is as well very promising because the cutoff value of IC_50_ is 1000 *μ*g/mL. The value higher than this indicates that the extract or other synthetic antioxidant is not effective as radical scavenger [29]. However, the scavenging effects of different parts of a plant might vary from each other due to the varied concentrations of active phytochemicals responsible for antioxidants in those parts [29]. Ascorbic acid is used as reference standard because ascorbic acid acts as a chain breaking scavenging agent that impairs the formation of free radicals in the process of intracellular substance formation throughout the body, including collagen, bone matrix, and tooth dentine [30].

The reducing capacity of a compound may serve as a significant indicator of its potential antioxidant activity [31]. The reducing power of* L. macrophylla* methanol extract along with that of ascorbic acid at concentrations between 50 and 500 *μ*g/mL showed that high absorbance indicates high reducing power [32]. The reducing power of the plant extract was increased as the amount of extract concentration increases. This is because the presence of reductants such as antioxidant substances in the samples causes the reduction of the Fe^3+^/ferricyanide complex to the ferrous form [33]. In our study, the reducing power of the fractions was lower than that of ascorbic acid but the IC_50_ value of chloroform fraction was very close to that of Ascorbic acid indicating that* L. macrophylla* has very statistically significant (*p* < 0.05) reducing power.

The scavenging activity of the extract against superoxide radical generated in the NaOH-alkaline DMSO-NBT system, resulting in the formation of the blue formazan, was studied in this research. The generated superoxide remains stable in solution, which reduces nitro blue tetrazolium into formazan dye at room temperature. Superoxide scavenger capable of reacting inhibits the formation of a red dye formazan [34]. The inhibition of formazan formation by the extract was reflected through the IC_50_ value for the fractions of the extracts. Ethyl acetate showed even lower IC_50_ value (252.35 *μ*g/mL) than that of the reference antioxidant ascorbic acid whereas chloroform extract has the IC_50_ value close to that of ascorbic acid. This finding demonstrates that* L. macrophylla* leaf extract is capable of nonenzymatically inhibiting the superoxide radical, produced in biological system, which is a precursor of many ROS and is shown to be harmful for various cellular components, although the enzyme superoxide dismutase possessed in aerobic and anaerobic organisms can catalyze the superoxide radical [35].

Orthosubstituted phenolic compounds were found more active than unsubstituted phenol. Hence, these compounds may exert prooxidant effect by interacting with Iron. O-Phenanthroline quantitatively forms complexes with Fe^2+^ which get disrupted in the presence of chelating agents [36]. Different fractions of the extract interfered with the formation of a ferrous-O-phenanthroline complex, thereby suggesting that the extract has metal chelating activity. In this research all the fractions were found to have better potential of iron chelating effects and the effects were higher than the reference antioxidative agent ascorbic acid. As iron plays a major role in the formation of lipid peroxidation in the body, the effects of antioxidant phytochemicals in the biological systems depend on their ability to scavenge radicals, chelate metals, activate the antioxidant enzymes, and inhibit the oxidases [37].

In this research CCl_4_ was administrated to cause oxidative stress in liver and the hepatic damage was associated with significant increased level of serum enzymatic and biochemical markers. This sort of hepatotoxicity by CCl_4_ is mainly due to the formation of the active metabolite trichloromethyl radical from CCl_4_ [38]. Covalent binding of this active metabolite to the macromolecules induces lipid peroxidation and forms lipid peroxides which produce damage to the membrane [39]. Rats treated with CCl_4_ developed significant hepatic damage as manifested by a significant increase in activities of AST, ALT, and ALP that are indicators of hepatocyte damage and loss of functional integrity. In treatment group extracts of* L. macrophylla* pointed toward normalization of liver cell functions by restoring serum AST, ALT, and ALP. We obtained significant increase in level of Bilirubin, CHL, LDL and significant decreased in level of HDL in CCl_4_ intoxicated liver samples of rat as compared to the normal control group. Damage of liver interrupted CHL, HDL, and LDL regulation. So level of CHL and LDL increased and HDL level decreased in CCl_4_ treated group.* L. macrophylla* showed hepatoprotective effect by restoring CHL, LDL, and HDL level. The oxidative damage observed in hepatic samples of rat with CCl_4_ treatment could be the consequence of hydrogen abstraction from membrane lipid molecules by H_2_O_2_-derived OH^*∙*^ and the failure of antioxidants to reestablish redox homeostasis [40, 41]. Lipid peroxidation, through a cascade mechanism, destroys membrane lipids and generates endogenous toxicants. These toxic molecules readily react with adjacent membrane proteins or DNA molecules which may cause more hepatic complications and functional abnormalities. However, phytochemical constituents possessing the radical scavenging effects can help the best neutralizing the free radicals responsible for lipid peroxidation. Other studies of our laboratory as well as published data revealed that* L. macrophylla* contains saponins, tannins, terpenoids, and flavonoids (isoquercetin, hyperoside, vitexin, myricetin, and kaempferol) [42]. These secondary metabolites exert antioxidant activities to scavenge free radicals and thereby prohibit lipid peroxidation [43, 44].

Histopathological studies also provided supportive evidence for biochemical analysis. Histology of the liver section of the normal control group showed that cytoplasm of the liver cells was well preserved, nucleus and nucleolus were prominent, and central veins were intact. Huge fatty changes, ballooning degeneration, necrosis, and broad infiltration of lymphocytes and kupffer cells around the central vein were showed in the liver sections of CCl_4_ intoxicated rats. Cellular boundaries were also lost in this group. The architectural pattern of liver sections of reference and treatment groups showed repairing lobular pattern with a mild to moderate degree of fatty change, necrosis, and lymphocytes infiltration which could be compared to the normal control group.

## 5. Conclusions

On the whole, it can be concluded that the altered biochemical profiles due to CCl_4_ exposure are reversed towards normalization by* L. macrophylla*. The contents of the extracts not only increased the regenerative and reparative capacity of the liver but, at the same time, prevented from oxidative damage. Beneficial effects of* L. macrophylla *illustrated in this study may be due to the presence of phytocomponents that have membrane stabilizing effects.

## Figures and Tables

**Table 1:** Preliminary phytochemical screening of different fractions of Hatikana extract.

Name of the test	Observation				
	n-HEx	MeEx	EtEx	CFEx	EAEx
Alkaloids	−−	−−	−−	−−	−−
Flavonoids	++	++	++	++	++
Steroids	++	++	++	++	++
Tannins	−	−	−	−	−
Saponins	+	−	−	+	+
Phlobatannins	−	+	+	−	−
Terpenoids	++	++	++	++	++

(+) and (++) indicate the intensity of phytochemical presence; (−) and (−−) similarly indicate their absence. Extracts (Ex) are, respectively, expressed as N-hexane (HE), methanol (ME), ethanol (Et), chloroform (CF), and ethyl acetate (EA).

**Table 2:** IC_50_ values of different organic fractions of *L. macrophylla* extract and standard agents.

Treatment	IC_50_ for different antioxidative measures			
	IC_50_ for DPPH	IC_50_ for FeCl_3_ reducing effect	IC_50_ for superoxide radical scavenging	IC_50_ for iron chelating effect
Curcumin	—	—	271.02	
Ascorbic acid (AA)	8.47	7.73		142.23
Methanol extract (MEEx)	55.46	122.74	287.08	118.02
Chloroform extract (CFEx)	2.64	15.24	274.16	68.55
Ethyl acetate extract (EAEx)	946.24	84.91	252.35	51.29

**Table 3:** Effect of *Leea macrophylla* (leaves) on total protein and CK-MB in CCl_4_-induced Wister rats.

Treatment	Group	Total protein (g/dL)	CK-MB (V/L)
Normal control (water)	Group I	4.63 ± 0.51	39.53 ± 15.50
Hepatic control (CCl_4_)	Group II	7.03 ± 0.15	94.67 ± 1.53
Reference control (silymarin)	Group III	4.23 ± 0.35	32.00 ± 2.00
Methanol fraction (100 mg/kg)	Group IV	5.73 ± 1.16	28.00 ± 12.12
Methanol fraction (200 mg/kg)	Group V	2.50 ± 0.20	197.67 ± 0.58
Chloroform fraction (100 mg/kg)	Group VI	3.97 ± 0.15	15.33 ± 2.52
Chloroform fraction (200 mg/kg)	Group VII	6.10 ± 0.10	38.00 ± 2.00
Ethyl acetate fraction (100 mg/kg)	Group VIII	6.50 ± 0.30	41.00 ± 3.00
Ethyl acetate fraction (200 mg/kg)	Group IX	22.00 ± 2.00	4.10 ± 0.02

## References

[1] Prakash T., Fadadu S. D., Sharma U. R. (2008). Hepatoprotective activity of leaves of Rhododendron arboreum in CCl_4_ induced hepatotoxicity in rats. *Journal of Medicinal Plants Research*.

[2] Wolf P. L. (1999). Biochemical diagnosis of liver disease. *Indian Journal of Clinical Biochemistry*.

[3] Loguercio C., Federico A. (2003). Oxidative stress in viral and alcoholic hepatitis. *Free Radical Biology & Medicine*.

[4] Aghel N., Rashidi I., Mombeini A. (2007). Hepatoprotective activity of *Capparis spinosa* root bark against CCl4 induced hepatic damage in mice. *Iranian Journal of Pharmaceutical Research*.

[5] Sehrawat A., Sultana S. (2006). Evaluation of possible mechanisms of protective role of *Tamarix gallica* against DEN initiated and 2-AAF promoted hepatocarcinogenesis in male Wistar rats. *Life Sciences*.

[6] Singh R. S., Singh A. N. (1981). On the identity and economic-medicaluses of Hastikarnapalsa (*Leea macrophylla* Roxb., Family Ampelidaceae) as evinced in the ancient texts and traditions. *Indian Journal of History of Science*.

[7] (2007). *Flora of China*.

[8] Chowdhary K. K., Singh M., Pillai U. (2008). Ethnobotanical survey of Rajasthan an update. *American-Eurasian Journal of Botany*.

[9] Uddin S. N. (2006). *Traditional Uses of Ethnomedicinal Plants of the Chittagong Hill Tracts*.

[10] Yusuf M., Wahab M. A., Yousuf M., Chowdhury J. U., Begum J. (2007). Some tribal medicinal plants of Chittagong Hill Tracts, Bangladesh. *Bangladesh Journal of Plant Taxonomy*.

[11] Uddin M. Z., Hassan M. A., Sultana M. (2006). Ethnobotanical survey of medicinal plants in Phulbari Upazila of Dinajpur district, Bangladesh. *Bangladesh Journal of Plant Taxonomy*.

[12] Islam M. B., Sarkar M. M. H., Shafique M. Z., Jalil M. A., Haque M. Z., Amin R. (2013). Phytochemical screening and anti-microbial activity studies on *Leea macrophylla* seed extracts. *Journal of Scientific Research*.

[13] Dewanjee S., Dua T. K., Sahu R. (2013). Potential anti-inflammatory effect of *Leea macrophylla* Roxb. leaves: a wild edible plant. *Food and Chemical Toxicology*.

[14] Mahmud Z. A., Bachar S. C., Qais N. (2011). Evaluation of anti- nociceptive activity and brine shrimp lethality bioassay of roots of *Leea macrophylla* Roxb. *International Journal of Pharmaceutical Sciences and Research*.

[15] Nizami A. N., Rahman M. A., Ahmed N. U., Islam M. S. (2012). Whole *Leea macrophylla* ethanolic extract normalizes kidney deposits and recovers renal impairments in an ethylene glycol-induced urolithiasis model of rats. *Asian Pacific Journal of Tropical Medicine*.

[16] Rahman M. A., Imran T. B., Islam S. (2013). Antioxidative, antimicrobial and cytotoxic effects of the phenolics of Leea indica leaf extract. *Saudi Journal of Biological Sciences*.

[17] Ibrahim M. A., Musa A. M., Aliyu A. B., Mayaki H. S., Gideon A., Islam M. S. (2013). Phenolics-rich fraction of Khaya senegalensis stem bark: antitrypanosomal activity and amelioratin of some parasite-induced pathological changes. *Pharmaceutical Biology*.

[18] Rincón C. T. S., Montoya J. E. Z., Gómez G. L. C. (2014). Optimizing the extraction of phenolic compounds from *Bixa orellana* L. and effect of physicochemical conditions on its antioxidant activity. *Journal of Medicinal Plants Research*.

[19] Trease G. E., Evans W. C. (1989). *Pharmacognosy*.

[20] Sanja S. D., Sheth N. R., Patel N. K., Patel D., Patel B. (2009). Characterization and evaluation of antioxidant activity of *Portulaca oleracea*. *International Journal of Pharmacy and Pharmaceutical Sciences*.

[21] Pandey M. M., Govindarajan R., Rawat A. K. S., Pushpangadan P. (2005). Free radical scavenging potential of *Saussarea costus*. *Acta Pharmaceutica*.

[22] Srinivasan R., Chandrasekar M. J. N., Nanjan M. J., Suresh B. (2007). Antioxidant activity of Caesalpinia digyna root. *Journal of Ethnopharmacology*.

[23] Reddy B. S., Reddy R. K. K., Reddy B. P., Ramakrishna S., Diwan P. V. (2008). Potential in vitro antioxidant and protective effects of *Soymida febrifuga* on ethanol induced oxidative damage in HepG2 cells. *Food and Chemical Toxicology*.

[24] Benzie I. F. F., Strain J. J. (1996). The ferric reducing ability of plasma (FRAP) as a measure of ‘antioxidant power’: the FRAP assay. *Analytical Biochemistry*.

[25] Zaoui A., Cherrah Y., Mahassini N., Alaoui K., Amarouch H., Hassar M. (2002). Acute and chronic toxicity of *Nigella sativa* fixed oil. *Phytomedicine*.

[26] Koleva I. I., van Beek T. A., Linssen J. P. H., de Groot A., Evstatieva L. N. (2002). Screening of plant extracts for antioxidant activity: a comparative study on three testing methods. *Phytochemical Analysis*.

[27] Suresh Kumar P. K., Sucheta S., Sudarshana Deepa V., Selvamani P., Latha S. (2008). Antioxidant activity in some selected Indian medicinal plants. *African Journal of Biotechnology*.

[28] Sarla S., Prakash M. A., Apeksha R., Subhash C. (2011). Free radical scavenging (DPPH) and ferric reducing ability (FRAP) of *Aphanamixis polystachya* (Wall) Parker. *International Journal of Drug Development and Research*.

[29] Chew A. L., Jessica J. J. A., Sasidharan S. (2012). Antioxidant and antibacterial activity of different parts of *Leucas aspera*. *Asian Pacific Journal of Tropical Biomedicine*.

[30] Aqil F., Ahmad I., Mehmood Z. (2006). Antioxidant and free radical scavenging properties of twelve traditionally used Indian medicinal plants. *Turkish Journal of Biology*.

[31] Meir S., Kanner J., Akiri B., Philosoph-Hadas S. (1995). Determination and involvement of aqueous reducing compounds in oxidative defense systems of various senescing leaves. *Journal of Agricultural and Food Chemistry*.

[32] Roy A., Khanra K., Mishra A., Bhattacharyya N. (2012). General analysis and antioxidant study of traditional fermented drink Handia, its concentrate and volatiles. *Advances in Life Science and its Applications*.

[33] Chung Y. C., Chang C. T., Chao W. W., Lin C. F., Chou S. T. (2002). Antioxidative activity and safety of the 50% ethanolic extract from red bean fermented by *Bacillus subtilis* IMR-NK1. *Journal of Agricultural and Food Chemistry*.

[34] Hagerman A. E., Riedl K. M., Jones G. A. (1998). High molecular weight plant polyphenolics (tannins) as biological antioxidants. *Journal of Agricultural and Food Chemistry*.

[35] Shirwaikar A., Punitha I. S. R. (2007). Antioxidant studies on the methanol stem extract of *Coscinium fenestration*. *Natural Product Sciences*.

[36] Mahakunakorn P., Tohda M., Murakami Y., Matsumoto K., Watanabe H. (2004). Antioxidant and free radical scavenging activity of Choto-san and its related constituents. *Biological & Pharmaceutical Bulletin*.

[37] Kulkarni A. P., Aradhya S. M., Divakar S. (2004). Isolation and identification of a radical scavenging antioxidant—punicalagin from pith and carpellary membrane of pomegranate fruit. *Food Chemistry*.

[38] Srivastava S. P., Chen N., Holtzman J. L. (1990). The in vitro NADPH-dependent inhibition by CCl4 of the ATP-dependent calcium uptake of hepatic microsomes from male rats: studies on the mechanism of the inactivation of the hepatic microsomal calcium pump by the CCL_3_. radical. *The Journal of Biological Chemistry*.

[39] Mujeeb M., Aeri V., Bagri P., Khan S. (2009). Hepatoprotective activity of the methanolic extract of *Tylophora indica* (Burm. f.) Merill. leaves. *International Journal of Green Pharmacy*.

[40] Nevin K. G., Vijayammal P. L. (2003). Effect of Aerva lanata on solid tumor induced by DLA cells in mice. *Fitoterapia*.

[41] Ohkawa H., Ohishi N., Yagi K. (1979). Assay for lipid peroxides in animal tissues by thiobarbituric acid reaction. *Analytical Biochemistry*.

[42] Islam A. M. T., Chowdhury M. A. U., Uddin M. E. (2013). Protective effect of methanolic extract of *Hylocereus polyrhizus* fruits on carbon tetra chloride-induced hepatotoxicity in rat. *European Journal of Medicinal Plants*.

[43] Sahreen S., Khan M. R., Khan R. A. (2010). Evaluation of antioxidant activities of various solvent extracts of *Carissa opaca* fruits. *Food Chemistry*.

[44] Aie B. M., Ladan M. J., Garba I. (2009). The modulatory effect of *Cochlospermum tinctorium* a rich aqueous root extract on liver damage induced by carbon tetrachloride in rats. *African Journal of Pharmacy and Pharmacology*.

